# Association of weight-adjusted waist index and body mass index with chronic low back pain in American adults: a retrospective cohort study and predictive model development based on machine learning algorithms (NHANES 2009–2010)

**DOI:** 10.3389/fpubh.2025.1617732

**Published:** 2025-07-11

**Authors:** Weiye Zhang, Yan Li, Pengwei Shao, Yuxuan Du, Ke Zhao, Jiawen Zhan, Lee A. Tan

**Affiliations:** ^1^China Academy of Chinese Medical Sciences Wangjing Hospital, Beijing, China; ^2^Department of Neurosurgery, University of California San Francisco, San Francisco, CA, United States; ^3^College of Management, Beijing University of Chinese Medicine, Beijing, China

**Keywords:** National Health and Nutrition Examination Survey, cross-sectional study, body mass index, weight-adjusted waist index, chronic low back pain

## Abstract

**Objective:**

This study utilized National Health and Nutrition Examination Survey (NHANES) data to investigate the associations between weight-adjusted waist index (WWI), body mass index (BMI), and chronic low back pain (CLBP) risk, and to develop machine learning models to assess the predictive capacity of WWI for CLBP.

**Methods:**

This cross-sectional analysis was based on NHANES 2009–2010 data. Weighted logistic regression models were employed to evaluate associations between WWI, BMI, and CLBP, with subgroup analyses, smooth curve fitting, and threshold effect analyses conducted to enhance result robustness. Receiver operating characteristic (ROC) curves were plotted to determine which indicator demonstrated stronger association with CLBP. Subsequently, permutation feature importance was applied for machine learning feature selection, random undersampling was utilized to address data imbalance, and the dataset was randomly divided into training and testing sets at a 7:3 ratio. Six machine learning algorithms were employed to predict CLBP occurrence and identify the optimal algorithm.

**Results:**

A total of 4,687 participants were included. Significant differences were observed between CLBP and non-CLBP groups in age, diabetes prevalence, smoking status, BMI, WWI, and education level. Both WWI and BMI showed significant associations with CLBP; after covariate adjustment, WWI demonstrated stronger and more consistent associations across quartiles. Subgroup analyses, nonlinear analyses, and ROC analyses further supported these findings. Machine learning feature selection identified 19 variables, with the Random Forest model demonstrating optimal performance.

**Conclusion:**

Both WWI and BMI were associated with increased CLBP risk, with WWI potentially serving as a more sensitive predictive indicator. Prospective studies are needed to validate causal relationships. The Random Forest machine learning model demonstrated high accuracy in CLBP prediction.

## 1 Introduction

Chronic low back pain (CLBP) is a highly prevalent condition occurring across all income levels—high, middle, and low—and affecting individuals of all ages, from children to older adults. Between 1990 and 2015, the global burden of disability-adjusted life years (DALYs) due to CLBP increased by 54%, making it one of the leading causes of disability worldwide (1). According to the chronic low back pain Research Standards Task Force of the U.S. National Institutes of Health, CLBP is defined as a back pain problem lasting at least 3 months and causing pain for at least half the time in the past 6 months (2). Preventing CLBP in high-risk populations is a critical challenge to address the substantial healthcare costs associated with treatment and rehabilitation (3). While factors such as obesity, smoking, and occupational risks have been linked to CLBP (4, 5), the relationship between CLBP, obesity, and obesity-related metrics remains unclear.

Increasing obesity has been associated with a higher risk of musculoskeletal disorders. Studies have shown that obesity can elevate fracture risk, partly due to increased estrogen levels in adipose tissue (6). Body mass index (BMI) and waist circumference (WC) are primary metrics for assessing obesity. BMI is the most widely used indicator, but it does not accurately differentiate obesity types or the distribution of fat. WC has been proposed as a more precise index for predicting obesity-related diseases than BMI, as it correlates strongly with abdominal fat imaging and is highly associated with cardiovascular disease (CVD) risk factors and mortality (7, 8). Limb fat distribution in CLBP patients tends to be higher than in non-CLBP patients, suggesting that obese CLBP patients require reduction of lower extremity adipose tissue (9). This indicates that indices focusing on fat distribution may be more appropriate for CLBP prediction. Commonly utilized anthropometric indicators for assessing abdominal obesity, such as waist-to-height or waist-to-weight ratios, can accurately reflect body fat percentage but cannot effectively reflect both fat and muscle mass components simultaneously. Increased adipose mass and decreased skeletal muscle mass may be associated with inflammation (10, 11), and the imbalanced ratio of fat to muscle mass represents an important contributing factor in CLBP development (12, 13).

In 2018, Park (14) introduced a novel obesity metric, the weight-adjusted waist index (WWI), which highlights the advantages of WC (15–18) and primarily reflects central obesity independent of body weight. Research has demonstrated significant associations between WWI and hypertension (19, 20), diabetes (21, 22), and cardiovascular diseases (23), among other conditions (24, 25). However, the relationship between CLBP risk, WWI, and BMI remains poorly understood.

Given this context, the present study aims to investigate the associations between WWI, BMI, and the risk of CLBP. Additionally, it seeks to explore potential factors influencing the relationship between WWI, BMI, and CLBP, which may hold significant implications for public health policies, prevention strategies, and patient education.

## 2 Materials and methods

### 2.1 Study design and population

This cross-sectional study utilized data from the 2009–2010 National Health and Nutrition Examination Survey (NHANES), available on the NHANES website.^1^ Details of NHANES’ continuous design are provided on the platform. Since responses related to chronic CLBP in the NHANES dataset are only available within the inflammatory arthritis questionnaire, which targets individuals aged 20 to 69 years, we included participants who completed this specific questionnaire for the assessment of CLBP. Exclusion criteria were: (1) missing CLBP data; (2) missing BMI values; (3) absent WC or weight measurements; and (4) pregnancy.

### 2.2 CLBP assessment

CLBP was identified based on NHANES criteria, defined as persistent pain in the region between the lower thoracic border and horizontal gluteal fold, lasting nearly daily for at least three consecutive months. Assessment required “yes” responses to the following questions: “Was there one time when you had pain, aching, or stiffness in your low back on almost every day for 3 or more months in a row?” and “Do you still have low back pain, aching, or stiffness?”

### 2.3 Study variables

The primary outcome was CLBP, while BMI and WWI served as independent variables. BMI was calculated as weight divided by the square of height (kg/m^2^), while WWI (cm/√kg) was derived by dividing WC (cm) by the square root of weight (kg).

### 2.4 Covariates

Demographic and clinical factors potentially influencing CLBP, BMI, and WWI were included as covariates: age, gender, education level, smoking status, alcohol consumption, poverty income ratio (PIR), sedentary time, diabetes status, and lumbar bone mineral density (BMD). Education was categorized into below high school, high school, or above high school. We referred to previous studies and categorized PIR into three groups: PIR < 1.5,1.5 ≤ PIR < 3.5, or PIR ≥ 3.5 (26). Alcohol consumption was defined as drinking more than 12 beverages in the past year, and smoking status as having smoked over 100 cigarettes in a lifetime. BMI was classified into three categories: <25 kg/m^2^, 25–30 kg/m^2^, and >30 kg/m^2^. Diabetes was based on a physician’s diagnosis, sedentary time (minutes/day) was self-reported, and lumbar BMD was measured via dual-energy X-ray absorptiometry (DXA).

### 2.5 Statistical analysis

Statistical analyses were conducted using R software (RStudio: Integrated Development for R. RStudio, PBC, Boston, MA, USA) (27, 28), with *p* < 0.05 considered statistically significant. Importing and visualizing datasets using the tidyverse package (29) in R, multiple imputation was performed using random forest methods via the mice package (30) to address missing data, generating five imputed datasets that incorporated all variables in the analysis. Results from the imputed datasets were combined for analysis. Characteristics of complete, missing, and imputed datasets were compared to ensure robustness. CDC-recommended sampling weights (“wtmec2yr”) were applied to reflect the U.S. population, following NHANES guidelines. Weighted means (± standard deviation) were used for continuous variables, and weighted percentages for categorical variables. Weighted logistic regression models were fitted using the survey package (31), and data manipulation was conducted using dplyr (32). T-tests assessed the relationships between BMI, WWI, and CLBP, and weighted logistic regression models were applied. Model 1 was unadjusted, Model 2 adjusted for gender, age, and education, and Model 3 adjusted for all covariates. Sensitivity analyses were performed to validate findings, including subgroup analyses, threshold effect analyses, smooth curve fitting by using the mgcv package (33). Log-likelihood ratio tests compared single-line and segmented models to identify thresholds. If nonlinear associations were observed, segmented regression models estimated effects and determined thresholds.

### 2.6 Machine learning algorithms

We conducted correlation tests for WWI and CLBP, demonstrating the high correlation between WWI and CLBP. However, we aim to accurately predict CLBP with the assistance of machine learning techniques, which has significant implications for the diagnosis and prevention of CLBP. To enhance the reliability of machine learning model training outcomes, we incorporated additional variables correlated with the previously included covariates, all derived from the 2009–2010 NHANES cycle dataset. These variables encompassed WWI-related measures (waist circumference and weight), BMD-related parameters (ward’s triangle BMD, femoral neck BMD, and lumbar spine BMD at L1-L4 vertebrae), Demographics-related factors (race, income level and marital status), and health status indicators (sleep quality, hypertension, healthy dietary patterns, and analgesic medication use), totaling 26 variables.

Python version 3.8 (Python Software Foundation, Wilmington, DE, USA) (34) is utilized for machine learning. The pandas (35) package was utilized for data analysis and processing. To conduct effective feature selection and enhance the interpretability of machine learning models, we employed permutation feature importance (PFI) (36) using the scikit-learn package (37). PFI quantifies feature importance by evaluating the increase in model prediction error following the permutation of feature values, thereby disrupting their relationship with the target variable. This severe imbalance may cause the model to be biased toward the majority class (no CLBP symptoms), thereby reducing its effectiveness in predicting CLBP. To mitigate this issue, we reduced the sample size of the majority class (no CLBP) to match the sample size in the minority class (CLBP). By employing the imblearn package (38) to implement random undersampling, we ensured balanced class representation during model training. This approach was selected due to its simplicity and effectiveness in improving model performance when handling imbalanced data.

All participants were randomly allocated into training and testing sets using a 7:3 ratio. We employed six machine learning algorithms implemented using the scikit-learn, LightGBM (39), and XGBoost (40) packages, including Random Forest, Gradient Boosting, Light Gradient Boosting Machine (LightGBM), Naive Bayes, Support Vector Machine (SVM), and Extreme Gradient Boosting (XGBoost).

The hyperparameter optimization process involves systematic adjustment of key parameters for each machine learning model. This study employs a grid search-based hyperparameter tuning approach combined with five-fold cross-validation to evaluate different parameter combinations, ensuring model stability and minimizing the influence of random variables. The objective is to identify optimal configurations that maximize model performance, with particular attention to metrics such as accuracy and area under the curve (AUC). The optimal model for depression prediction was determined by comparing performance metrics on the test set.

## 3 Results

### 3.1 Basic clinical characteristics

**Figure 1:**
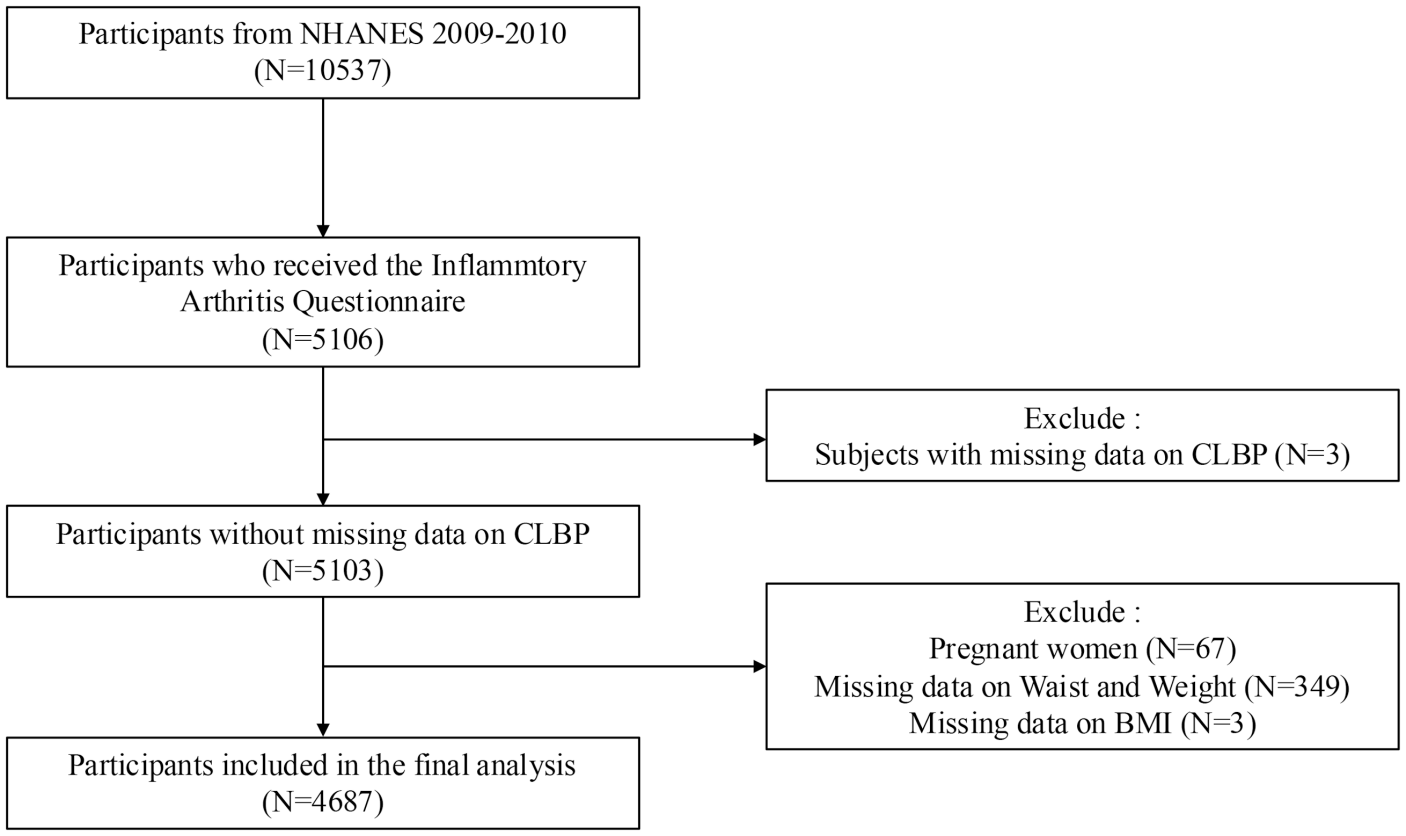
Screening process for participant inclusion. NHANES: National Health and Nutrition Examination Survey.

Out of 10,537 participants from the 2009–2010 NHANES dataset, 4,687 participants were included after excluding individuals with missing CLBP data (*n* = 3), BMI data (*n* = 3), waist and weight data (*n* = 349), and pregnant women (*n* = 67). The screening process is shown in Figure 1.

Table 1 compares the characteristics of participants with CLBP (*n* = 566) and those without CLBP (*n* = 4,121). Significant differences were observed in age, with CLBP individuals being older (47.35 years vs. 43.54 years, *p* < 0.001). The prevalence of diabetes was significantly higher in the CLBP group (15.91% vs. 8.66%, *p* < 0.001). Smokers were more prevalent among CLBP participants (62.37%) compared to the non-CLBP group (42.88%, *p* < 0.001). Both BMI and WWI were higher in the CLBP group (BMI: *p* < 0.001; WWI: 11.13 ± 0.80 vs. 10.88 ± 0.80, *p* < 0.001). Education level also differed significantly, with more participants having less than high school education in the CLBP group (*p* = 0.014). No significant differences were observed in sedentary time, total spine BMD, drinking status, or sex distribution.

**Table 1:** Baseline characteristics.

Variable	Non-CLBP ^#^ *n* = 4,121	CLBP *n* = 566	*p*-value
AGE (years)	43.54 ± 14.11	47.35 ± 13.21	<0.001
Sex (%)			0.089
Male	2043 (49.58%)	259 (45.76%)	
Female	2078 (50.42%)	307 (54.24%)	
Education (%)			0.014
Less than high school	1,092 (26.56%)	173 (30.57%)	
High school	937 (22.79%)	143 (25.27%)	
More than high school	2083 (50.66%)	250 (44.17%)	
Diabetes (%)			<0.001
Diabetes	351 (8.66%)	88 (15.91%)	
Non-diabetes	3,701 (91.34%)	465 (84.09%)	
Drinking status (%)			0.179
Drinking	2,831 (75.94%)	415 (78.60%)	
Non-drinking	897 (24.06%)	113 (21.40%)	
Smoking status (%)			<0.001
Non-smoking	2,354 (57.12%)	213 (37.63%)	
Smoking	1767 (42.88%)	353 (62.37%)	
PIR (%)			<0.001
0–1.3	1,266 (33.99%)	216 (41.06%)	
1.3–3.5	1,320 (35.44%)	186 (35.36%)	
>3.5	1,139 (30.58%)	124 (23.57%)	
BMI (kg/m^2^)	28.92 ± 6.61	30.85 ± 7.90	<0.001
BMI			<0.001
0–25	1,207 (29.29%)	125 (22.08%)	
25–30	1,388 (33.68%)	172 (30.39%)	
>30	1,526 (37.03%)	269 (47.53%)	
WWI (cm/√kg)	10.88 ± 0.80	11.13 ± 0.80	<0.001
WWI			<0.001
8.42–10.35	1,071 (25.99%)	100 (17.67%)	
10.35–10.90	1,056 (25.62%)	116 (20.49%)	
10.90–11.43	1,014 (24.61%)	158 (27.92%)	
11.43–13.82	980 (23.78%)	192 (33.92%)	
Sedentary (min)	313.04 ± 196.27	326.74 ± 201.60	0.121
Total spine BMD	1.04 ± 0.14	1.04 ± 0.14	0.585

# Unweighted number. BMI, body mass index; WWI, weight-adjusted waist index; CLBP, chronic low back pain; BMD, bone mineral density; PIR, Personal Income Ratio.

### 3.2 Association between WWI, BMI, and CLBP

Table 2 displays the associations between WWI, BMI, and CLBP across three models. For WWI, each unit increase was consistently associated with higher odds of CLBP, even after full adjustment for all covariates (Model 3: OR = 1.31, 95% CI: 1.08–1.60, *p* = 0.006). Quartiles of WWI also showed a significant trend, with participants in Quartile 4 having the highest odds of CLBP compared to Quartile 1 (Model 3: OR = 1.62, 95% CI: 1.12–2.34, *p* = 0.010).

**Table 2:** Association between WWI, BMI, and CLBP.

Mode	Model 1		Model 2		Model 3	
VAR	OR (95%CI)	*P*-value	OR (95%CI)	*P*-value	OR (95%CI)	*P*-value
WWI (continuous variable) (cm/√kg)	1.54 (1.29–1.85)	<0.0001	1.40 (1.13–1.73)	0.002	1.31(1.08–1.60)	0.006
WWI (categorical variable)						
Quartile 1	Ref	Ref	Ref	Ref	Ref	Ref
Quartile 2	1.08 (0.84–1.39)	0.531	0.98 (0.77–1.26)	0.902	0.97 (0.76–1.25)	0.822
Quartile 3	1.71 (1.33–2.22)	<0.0001	1.46 (1.10–1.96)	0.010	1.43 (1.07–1.92)	0.015
Quartile 4	2.31 (1.64–3.28)	<0.0001	1.84 (1.24–2.73)	0.002	1.62 (1.12–2.34)	0.010
*P* for trend	1.35 (1.20–1.53)	<0.0001	1.26 (1.10–1.44)	0.002	1.21 (1.06–1.37)	0.004
BMI (continuous variable) (kg/m^2^)	1.04 (1.02–1.06)	<0.0001	1.03 (1.01–1.05)	0.001	1.03 (1.01–1.05)	0.006
BMI (categorical variable)						
Quartile 1	Ref	Ref	Ref	Ref	Ref	Ref
Quartile 2	1.31 (1.07–1.59)	0.009	1.20 (0.96–1.49)	0.107	1.20 (0.95–1.51)	0.130
Quartile 3	1.72 (1.23–2.41)	<0.0001	1.55 (1.09–2.00)	0.002	1.45 (1.08–1.95)	0.013
*P* for trend	1.31 (1.13–1.52)	<0.0001	1.25 (1.09–1.44)	0.002	1.21 (1.03–1.40)	0.017

Model 1 was crude model. Model 2 adjusting age, sex, education level. Model 3 adjusting all covariates. WWI (categorical variable), Quartile 1: 8.42–10.35; Quartile 2: 10.35–10.90; Quartile 3: 10.90–11.43; Quartile 4: 11.43–13.82; BMI (categorical variable), Quartile 1:0–25; Quartile 2: 25–30; Quartile 3: >30; Ref, reference; OR, odds ratio; CI, confidence interval; BMI, body mass index; WWI, weight-adjusted waist index; CLBP, chronic low back pain.

For BMI, each unit increase was associated with a smaller, yet significant, increase in CLBP risk (Model 3: OR = 1.03, 95% CI: 1.01–1.05, *p* = 0.006). While Quartile 3 showed significant associations across models, Quartile 4 results were less consistent. Overall, WWI demonstrated a stronger and more consistent relationship with CLBP than BMI, suggesting its potential as a better predictor of CLBP.

### 3.3 Subgroup analysis

Subgroup analyses (Table 3) revealed positive associations between WWI, BMI, and CLBP across various demographic and health-related groups, including age, sex, education, smoking, and diabetes. WWI had a stronger effect size in individuals aged 40–60 years, males, and those with lower education levels. BMI associations were notable among individuals with diabetes or lower BMD. No significant interaction effects were observed, confirming the robustness of the findings.

**Table 3:** Subgroup analysis of the relationship between WWI, BMI, and CLBP.

Groups	WWI (continuous)		BMI (continuous)	
	Model 3 OR (95%CI)	P for interaction	Model 3 OR (95%CI)	P for interaction
Age (year)		0.1041		0.1459
20–40	1.16 (0.89, 1.53)		1.01 (0.98, 1.04)	
40–60	1.54 (1.32, 1.80)		1.04 (1.02, 1.06)	
>60	1.16 (0.75, 1.80)		1.03 (0.98, 1.07)	
Gender		0.4947		0.1635
Male	1.37 (1.13, 1.67)		1.02 (1.00, 1.04)	
Female	1.27 (1.01, 1.61)		1.03 (1.01, 1.06)	
Education		0.5145		0.2200
Less than high school	1.43 (1.05, 1.95)		1.05 (1.00, 1.09)	
High school	1.49 (1.10, 2.01)		1.04 (1.01, 1.08)	
More than high school	1.18 (0.90, 1.54)		1.01 (0.99, 1.03)	
Smoking status		0.6684		0.8183
Non-smoking	1.27 (0.96, 1.69)		1.03 (1.00, 1.05)	
Smoking	1.35 (1.11, 1.63)		1.03 (1.00, 1.06)	
Drinking status		0.1628		0.4056
Drinking	1.37 (1.10, 1.70)		1.02 (1.00, 1.05)	
Non-drinking	1.18 (0.96, 1.46)		1.03 (1.01, 1.06)	
Diabetes		0.7321		0.0549
Diabetes	1.24 (0.85, 1.82)		1.07 (1.03, 1.12)	
Non-diabetes	1.32 (1.08, 1.62)		1.02 (1.00, 1.04)	
PIR		0.0079		0.2998
0–1.3	1.67 (1.29, 2.16)		1.05 (1.01, 1.08)	
1.3–3.5	1.09 (0.86, 1.38)		1.02 (0.98, 1.05)	
>3.5	1.37 (1.03, 1.83)		1.02 (0.99, 1.05)	
Total spine BMD		0.8257		0.0036
Low	1.34 (1.02, 1.76)		1.05 (1.01, 1.09)	
Middle	1.21 (0.91, 1.62)		1.01 (0.99, 1.04)	
High	1.39 (1.00, 1.92)		1.04 (1.01, 1.06)	

Model 3 adjusting all covariates. Total spine BMD: Low (0.85–0.95); Middle (1.02–1.06); High (1.12–1.23); Ref, reference; OR, odds ratio; CI, confidence interval; BMI, body mass index; WWI, weight-adjusted waist index.

### 3.4 Threshold effect analysis

**Figure 2:**
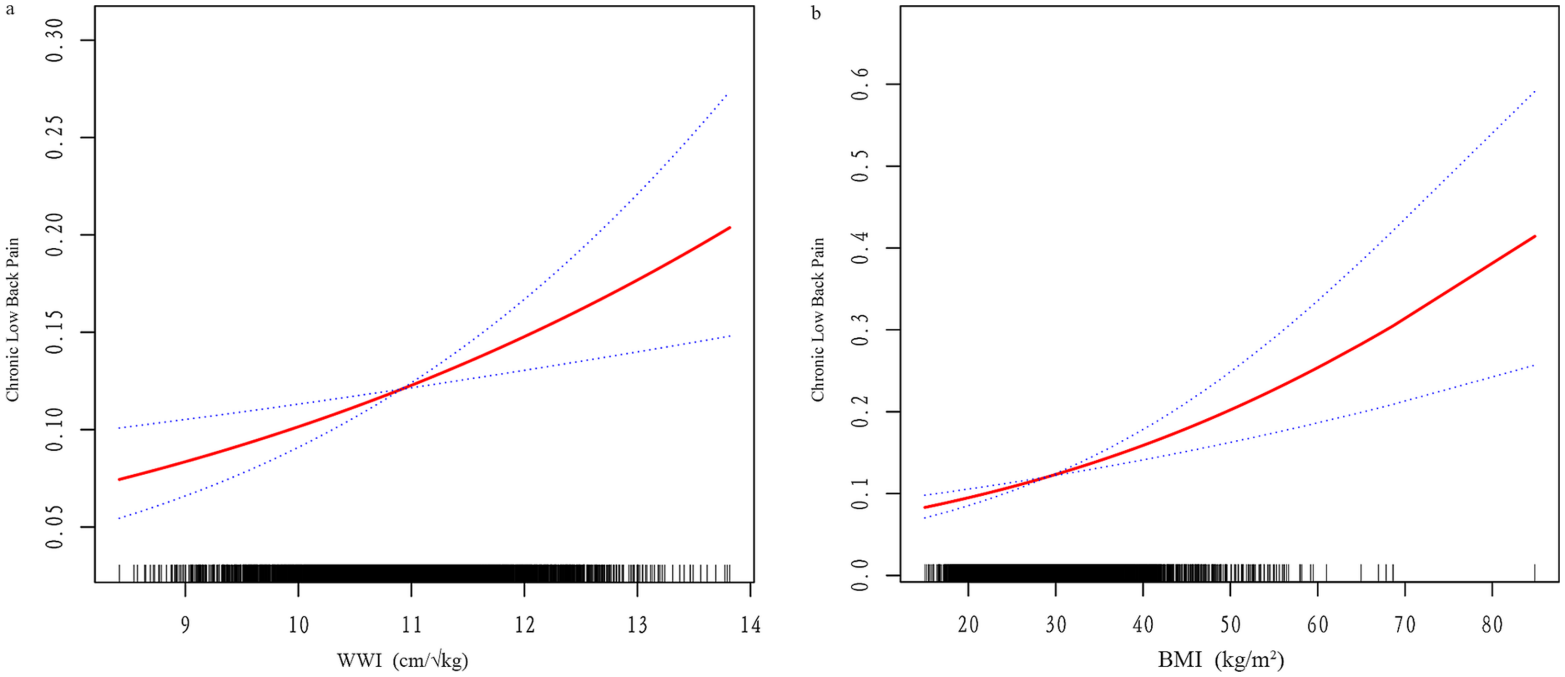
**(a)** Smooth curve fitting of WWI and CLBP; **(b)** Smooth curve fitting of BMI and CLBP. The red line represents the fitted curve, and blue lines indicate the 95% confidence interval.

Smooth curve fitting and threshold effect analysis (Figure 2; Table 4) identified nonlinear relationships between WWI, BMI, and CLBP. For BMI, a threshold was identified at 20.17, where BMI had a negative association with CLBP below this value (*β* = 0.84, 95% CI: 0.76–0.93) and a positive association above it (*β* = 1.03, 95% CI: 1.03–1.04). For WWI, the threshold was 11.6, with a significant positive association below the threshold (*β* = 1.31, 95% CI: 1.21–1.42), while no significant relationship was observed above the threshold.

**Table 4:** Threshold effect analysis of the relationship among WWI, BMI, and CLBP.

Categories	WWI	*p*-value	BMI	*p*-value
Linear effect model	1.24 (1.17, 1.31)	<0.0001	1.03 (1.02, 1.04)	<0.0001
Non-linear effect model				
Infection point (K)	11.6		20.17	
< K, effect 1	1.31 (1.21, 1.42)	<0.0001	0.84 (0.76, 0.93)	0.0009
> K, effect 2	1.07 (0.92, 1.25)	0.3812	1.03 (1.03, 1.04)	<0.0001
Effect difference between 2 and 1	0.82 (0.67, 0.99)	0.0418	1.22 (1.11, 1.35)	<0.0001
Log-likelihood ratio	0.04		<0.001	

BMI, body mass index; WWI, body mass index; p, *p*-value; Threshold effect analysis adjusted all variables in Table 1.

### 3.5 ROC curve analysis between BMI and WWI

**Figure 3:**
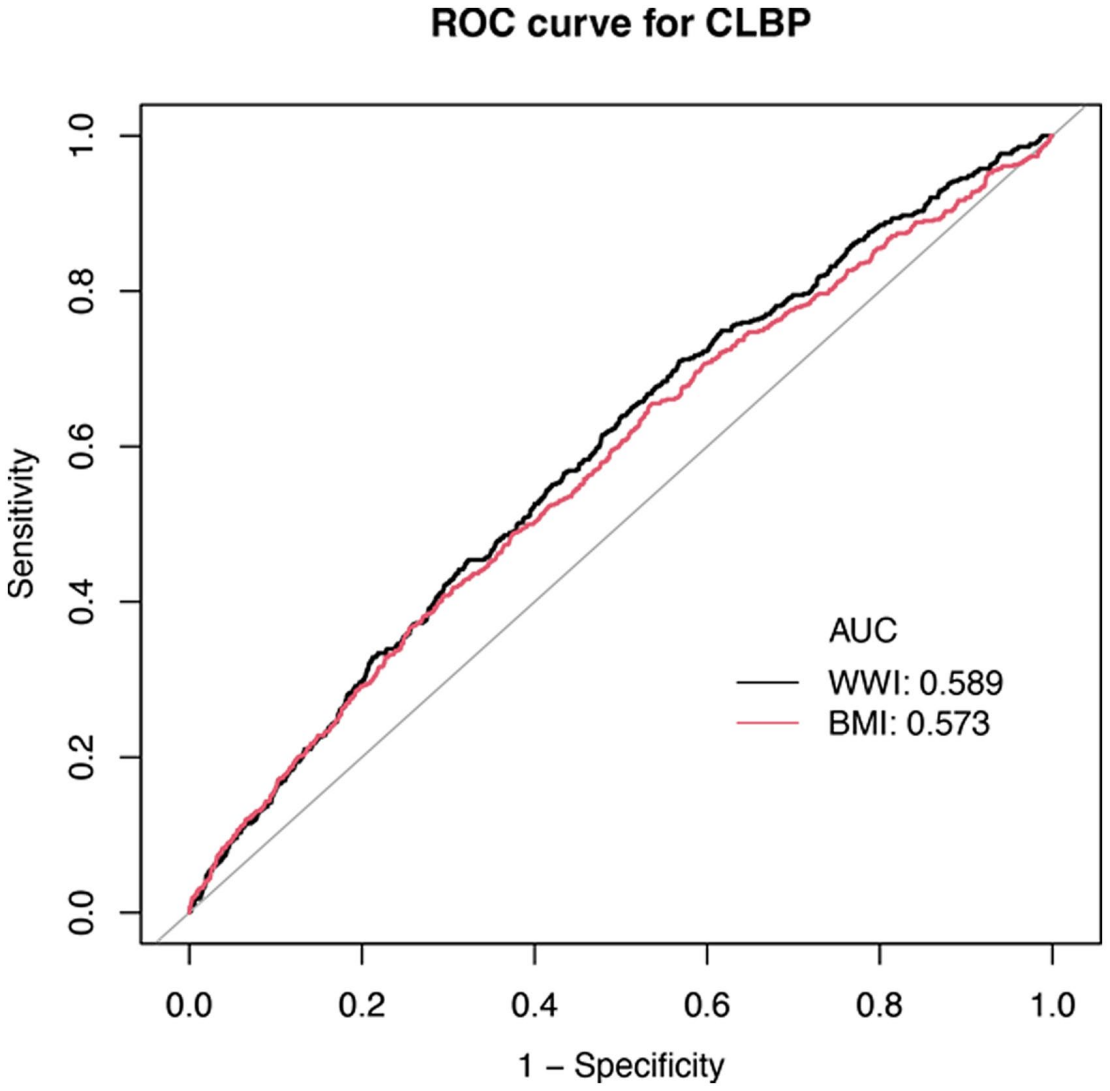
ROC curve for WWI, BMI, and CLBP. BMI, Body Mass Index; WWI, Waist-to-Weight index. CLBP, Chronic Low Back Pain.

Compared to BMI, WWI demonstrates slightly higher diagnostic performance, with an AUC of 0.589 versus 0.573 for BMI (Figure 3). However, both indicators exhibit limited discriminative ability, as their ROC curves lie relatively close to the diagonal. Nevertheless, WWI appears to be a marginally more promising indicator for CLBP, offering slightly better predictive value than BMI. Therefore, we applied machine learning methods to further evaluate the predictive capability of WWI for CLBP.

### 3.6 Machine learning model performance comparison

#### 3.6.1 Machine learning feature selection

We employed PFI to screen the 26 included variables, selecting 19 variables with positive perm importance mean values (analgesic medication use, waist circumference, weight, sleep quality, ward’s triangle BMD, WWI, education, smoking status, hypertension, income level, sedentary, diabetes, PIR, L2BMD, marital status, BMI, sex, L4BMD, femoral neck BMD) for inclusion in the machine learning models. Detailed results of PFI feature selection are provided in Supplementary file.

#### 3.6.2 Machine learning algorithm performance comparison

**Figure 4:**
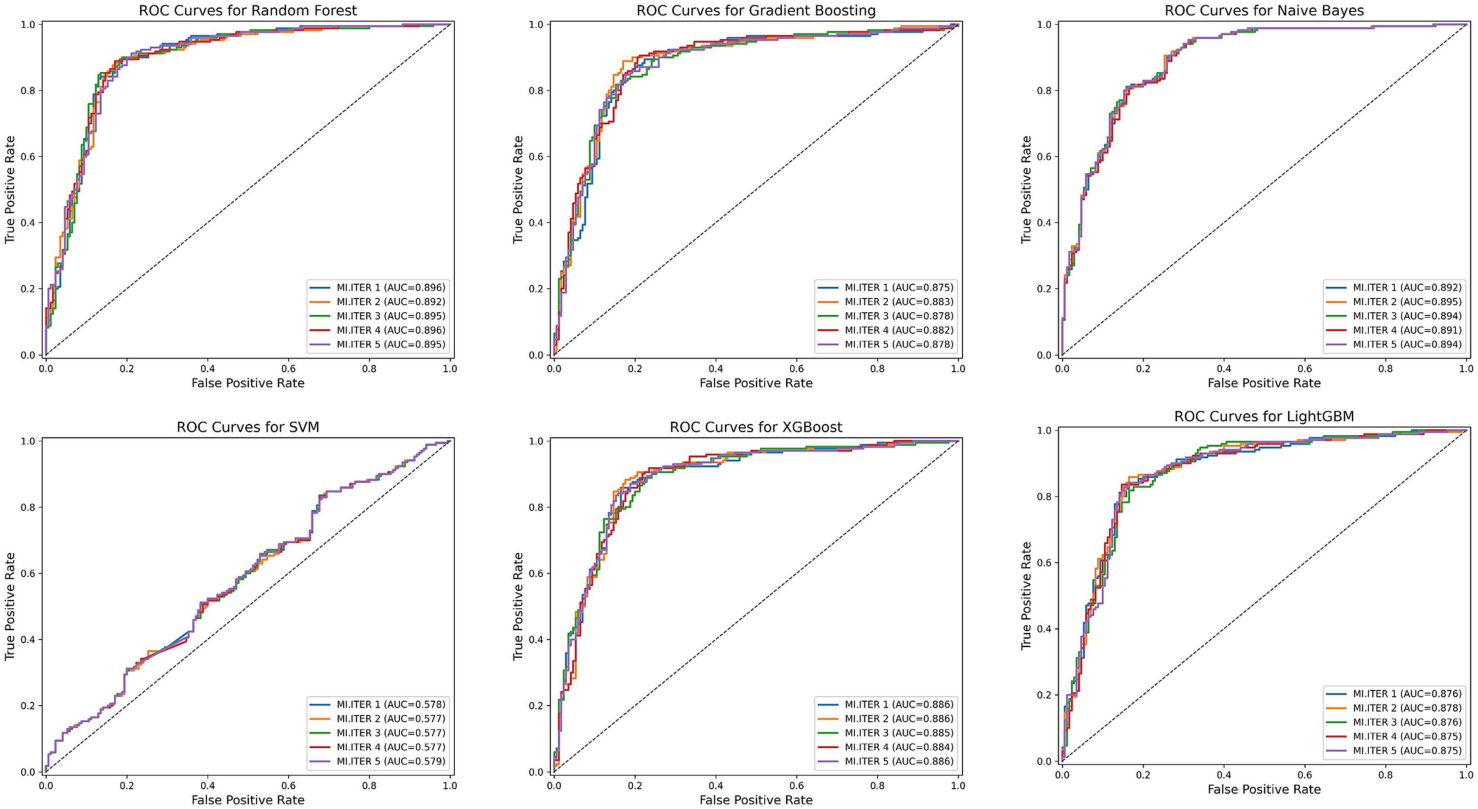
The ROC curves for the six machine learning models. MI, ITER represents the 5 new datasets following multiple imputation.

Six machine learning models (Random Forest, Gradient Boosting, LightGBM, Naive Bayes, SVM, XGBoost) were applied to the training set for model training, and the test set was used to evaluate the predictive performance of these models. Since multiple imputation via random forest methods was employed for missing value imputation, we compared the mean values across the 5 imputed datasets. Figure 4 shows the receiver operating characteristic (ROC) curves for machine learning models predicting CLBP.

Among all model test results, Random Forest consistently achieved the highest metrics across all categories, indicating robust overall performance. The Random Forest model achieved the best overall performance, with the highest accuracy (0.85), excellent sensitivity (0.88), and strong specificity (0.82), indicating a well-balanced ability to identify both positive and negative cases. It also had the highest F1-score (0.85), reflecting a strong balance between precision and recall. Moreover, its AUC was the highest (0.89), demonstrating outstanding discriminatory power. Naive Bayes also performed well, with good accuracy (0.81), balanced sensitivity (0.81) and specificity (0.80), and a solid F1-score (0.81). Its AUC (0.89) was nearly comparable to Random Forest, with a slightly narrower confidence interval, suggesting stable predictive power. XGBoost showed strong performance as well, with high accuracy (0.83), good sensitivity (0.85), and specificity (0.81). Table 5 presents detailed test results for all machine learning models using the test set.

**Table 5:** Performance metrics of different machine learning approaches.

Model	Accuracy	Precision	Sensitivity	Specificity	F1 Score	AUC (95% CI)
Random Forest	0.846	0.827	0.876	0.816	0.851	0.894 (0.855–0.927)
Naive Bayes	0.806	0.802	0.813	0.799	0.807	0.893 (0.857–0.923)
XGBoost	0.831	0.818	0.852	0.811	0.835	0.883 (0.842–0.917)
Gradient Boosting	0.828	0.812	0.854	0.802	0.833	0.876 (0.835–0.912)
LightGBM	0.811	0.803	0.825	0.798	0.814	0.871 (0.830–0.906)
SVM	0.549	0.601	0.293	0.806	0.394	0.551 (0.493–0.609)

## 4 Discussion

This cross-sectional study of 4,687 participants examined the associations between WWI, BMI, and CLBP risk. Both higher WWI and BMI were significantly associated with increased CLBP risk. Furthermore, WWI demonstrated a stronger and more consistent association with CLBP risk across quartiles, suggesting it may serve as a more sensitive body composition indicator for predicting chronic low back pain. Subgroup analyses revealed positive associations between WWI, BMI, and CLBP across various age groups, genders, education levels, and health-related subgroups. No significant interaction effects were observed among these variables, supporting the robustness of these findings. Smooth curve fitting and threshold effect analyses identified nonlinear relationships between WWI, BMI, and CLBP, with thresholds at BMI 20.17 and WWI 11.6. The associations with CLBP exhibited different characteristics on either side of these thresholds, suggesting potential underlying mechanisms influencing WWI and BMI effects on CLBP near these critical points.

CLBP represents a cardinal manifestation of numerous conditions, including lumbar disc herniation, spinal stenosis, and spondylolisthesis, warranting substantial clinical and patient attention. Managing these underlying pathologies presents considerable therapeutic challenges (41–43). Prolonged CLBP is closely linked to a decline in quality of life, reduced work capacity, and higher prevalence of mental health disorders (44, 45). Moreover, research suggests that CLBP may contribute to the acceleration of biological aging (46). The extensive use of analgesics resulting from CLBP is associated with increased healthcare utilization among individuals with severe CLBP (47, 48).

Obesity and overweight have been confirmed as major risk factors for various diseases. The World Health Organization (WHO) defines obesity as excessive fat accumulation beyond normal physiological needs, caused by a long-term imbalance between caloric intake and energy expenditure (49). Factors such as slow metabolism, reduced physical activity, dietary imbalances, and life stress contribute to the increased risk of obesity (50). A study involving 4,289 participants demonstrated a significant association between obesity and increased risk of CLBP (51). Both metabolically healthy and unhealthy obesity were shown to significantly increase the risk of joint pain and low back pain (52). This highlights the critical role of regional fat distribution in the pathogenesis of obesity-related diseases, although few studies have explored how fat distribution impacts CLBP (9).

The emergence of the WWI index addresses the limitations of traditional body mass index (BMI) in obesity assessment, thereby more accurately reflecting intracorporeal fat distribution. Different fat distribution patterns reflect biomechanical stress in various body regions, while BMI cannot differentiate between muscle mass and fat mass. Recent studies have increasingly demonstrated the association between WWI and skeletal muscle. WWI can estimate fat and muscle mass, potentially influencing bone health (53). In community-dwelling adults, higher WWI values were associated with adverse body composition outcomes, indicating high fat mass, low muscle mass, and low bone mass. WWI has been proven to predict early deterioration of trabecular bone structure (54). Higher WWI was associated with increased prevalence of hip and spinal fractures (55). Furthermore, WWI is applicable for predicting metabolic risk, correlating with diabetes and cardiovascular disease risk and mortality (56). WWI can also predict renal function and compared to BMI, contributes to better patient prevention and treatment strategies (57). Emerging evidence suggests a potential association between chronic pain and increased adiposity. Women with larger hip or waist circumferences exhibit a significantly higher prevalence of chronic pain, which is strongly correlated with elevated levels of inflammatory biomarkers such as IL-6 (58).

The prediction of CLBP can serve as a valuable indicator for at-risk populations. We employed six machine learning algorithms to predict the occurrence of CLBP, aiming to help with early prevention efforts. Ultimately, we found that the Random Forest model was the most suitable predictive model. Additionally, we found that Naive Bayes and XGBoost also demonstrated excellent predictive performance, with strong results across evaluation metrics including F1-score, accuracy, and sensitivity, thereby confirming that the variables included in this study show significant potential for CLBP prediction.

This study has several strengths. First, it used a large population dataset to investigate the relationships between WWI, BMI, and CLBP. The data were derived from an authoritative database, making the results representative of the general U.S. population. In addition, the study incorporated factors such as lumbar spine BMD, diabetes, smoking, alcohol consumption, and poverty levels, addressing potential confounders related to CLBP and ensuring the reliability of the findings. However, there are limitations. First, the NHANES database included CLBP data only from the 2009–2010 cycle, limiting the sample size. Second, NHANES data represent the U.S. population only, restricting the study’s geographical scope. Third, CLBP was self-reported through questionnaires, which may introduce some measurement errors. In the future, we will conduct in-depth research on other chronic pain conditions, such as thoracic spine pain (59), headaches, cervical spine pain (60), etc.

## 5 Conclusion

This study found a significant association between WWI and CLBP, and the Random Forest machine learning model demonstrated good predictive performance for CLBP.

## Data Availability

Publicly available datasets were analyzed in this study. This data can be found here: www.cdc.gov/nchs/nhanes/index.html.

## Footnotes

^1^ https://www.cdc.gov/nchs/nhanes
