# Supplementary material for: Association of weight-adjusted waist index and body mass index with chronic low back pain in American adults: a retrospective cohort study and predictive model development based on machine learning algorithms (NHANES 2009–2010)
Source: Front Public Health. 2025 Jul 11;13:1617732. doi: 10.3389/fpubh.2025.1617732 (PMC12289706; doi:10.3389/fpubh.2025.1617732)
Supplement: Supplementary file 1 [file Table_1.docx]

Supplementary Material

**Supplementary Table 1 Missing rate of variables**

| Variables | Miss rate (%) |
| --- | --- |
| AGE | 0 |
| Sedentary | 0.21 |
| Total spine BMD | 28.01 |
| Sex | 0 |
| Education | 0.19 |
| Diabetes | 1.75 |
| Drinking status | 9.2 |
| Smoking status | 0 |
| PIR | 9.3 |
| BMI | 0 |
| WWI | 0 |

Abbreviations: BMI, body mass index; WWI, weight-adjusted waist index; CLBP, chronic low back pain; BMD, bone mineral density; PIR, Personal Income Ratio

**Supplementary Table 2 Summary statistics of complete cases, missing data and imputed mode**

| Variables | Complete Cases Data | | Missing_Data | | Imputed_model_Data | |
| --- | --- | --- | --- | --- | --- | --- |
|  | Mean | SD | Mean | SD | Mean | SD |
| AGE | 44.00 | 14.06 | 46.95 | 13.89 | 44.00 | 14.06 |
| Sex | 1.51 | 0.50 | 1.51 | 0.50 | 1.51 | 0.50 |
| PIR | 2.44 | 1.66 | 2.42 | 1.66 | 2.39 | 1.60 |
| Sedentary | 314.69 | 196.95 | 315.20 | 196.20 | 314.76 | 196.75 |
| Education | 2.23 | 0.85 | 2.15 | 0.86 | 2.23 | 0.85 |
| Diabetes | 1.90 | 0.29 | 1.88 | 0.33 | 1.90 | 0.29 |
| Drinking status | 1.24 | 0.43 | 1.23 | 0.42 | 1.31 | 0.46 |
| Smoking status | 0.45 | 0.50 | 0.48 | 0.50 | 0.45 | 0.50 |
| BMI | 29.16 | 6.80 | 30.48 | 7.73 | 29.16 | 6.80 |
| WWI | 10.91 | 0.81 | 11.10 | 0.79 | 10.91 | 0.81 |
| Total spine BMD | 1.04 | 0.14 | 1.02 | 0.15 | 1.04 | 0.14 |
|  |  |  |  |  |  |  |
| Variables | Complete Cases Data | | Missing_Data | | Imputed_model_Data | |
|  | Min | Max | Min | Max | Min | Max |
| AGE | 20.00 | 69.00 | 20.00 | 69.00 | 20.00 | 69.00 |
| Sex | 1.00 | 2.00 | 1.00 | 2.00 | 1.00 | 2.00 |
| PIR | 0.00 | 5.00 | 0.00 | 5.00 | 0.00 | 5.00 |
| Sedentary | 0.00 | 1200.00 | 0.00 | 1080.00 | 0.00 | 1200.00 |
| Education | 1.00 | 3.00 | 1.00 | 3.00 | 1.00 | 3.00 |
| Diabetes | 1.00 | 2.00 | 1.00 | 2.00 | 1.00 | 2.00 |
| Drinking status | 1.00 | 2.00 | 1.00 | 2.00 | 1.00 | 2.00 |
| Smoking status | 0.00 | 1.00 | 0.00 | 1.00 | 0.00 | 1.00 |
| BMI | 15.02 | 84.87 | 15.22 | 84.87 | 15.02 | 84.87 |
| WWI | 8.42 | 13.82 | 8.58 | 13.82 | 8.42 | 13.82 |
| Total spine BMD | 0.53 | 1.77 | 0.54 | 1.51 | 0.53 | 1.77 |

Abbreviations: BMI, body mass index; WWI, weight-adjusted waist index; CLBP, chronic low back pain; BMD, bone mineral density; PIR, Personal Income Ratio

**Supplementary Table 3: Result of permutation feature importance**

| Feature | Perm_importance_mean |
| --- | --- |
| analgesic medication use | 0.25726 |
| waist circumference | 0.10222 |
| Weight | 0.01986 |
| sleep quality | 0.00957 |
| ward's triangle BMD | 0.00409 |
| WWI | 0.00407 |
| education | 0.00265 |
| Smoking status | 0.00242 |
| hypertension | 0.00146 |
| income level | 0.00145 |
| sedentary | 0.00102 |
| Diabetes | 0.00083 |
| PIR | 0.00070 |
| L2BMD | 0.00066 |
| marital status | 0.00030 |
| BMI | 0.00023 |
| Sex | 0.00015 |
| L4BMD | 0.00003 |
| femoral neck BMD | 0.00003 |
| L3BMD | -0.00005 |
| total spine BMD | -0.00009 |
| L1BMD | -0.00011 |
| drinking status | -0.00021 |
| healthy diet | -0.00037 |
| race | -0.00167 |
| age | -0.00211 |
